# Baseline monocyte and its classical subtype may predict efficacy of PD-1/PD-L1 inhibitor in cancers

**DOI:** 10.1042/BSR20202613

**Published:** 2021-01-29

**Authors:** Yilin Shao, Shuchen Lin, Ping Zhang, Jian Zhang, Dongmei Ji, Zhonghua Tao, Xichun Hu

**Affiliations:** 1 Department of Medical Oncology, Fudan University Shanghai Cancer Center, Shanghai Medical College, Fudan University, Shanghai 200032, China; 2 Cancer Institute of Fudan University Shanghai Cancer Center, Shanghai Medical College, Fudan University, Shanghai 200032, China

**Keywords:** PD-1/PD-L1 inhibitor, predictive factor, response

## Abstract

Background: Programmed death 1 (PD-1)/ programmed death-ligand 1 (PD-L1) inhibitor is one of the most popular immune therapies. Biomarkers for predicting response are highly needed, but no biomarkers are widely used till now.

Patients and methods: From February 2018 to April 2019, pan-cancer patients treated with PD-1 or PD-L1 inhibitor as a single agent in our center were included. The benefit group included patients with partial response, complete response and stable disease, while the patients with progressive disease were classified into the nonbenefit group, according to the RECIST 1.1 criteria. Baseline peripheral blood was sampled to determine absolute monocyte count (AMC) and/or classical monocyte frequency (CMF) of peripheral blood mononuclear cells. Then, the association of the above-mentioned two biomarkers with response or progression-free survival (PFS) was evaluated.

Results: In total, 107 patients enrolled in the present study. The nonbenefit group had significantly larger number of AMC than benefit group (*P*<0.001), and patients with higher AMC had decreased PFS time (*P*=0.001). Of 39 patients tested for CMF, the nonbenefit group had significantly higher CMF than benefit group (*P*=0.002), and patients with higher CMF had significantly decreased PFS time (*P*=0.002). The sensitivity of AMC and CMF was 87.9% and 85.7%, respectively, and the specificity was 44.9% and 61.1%, respectively. Multivariate analysis showed high baseline CMF and AMC were both significantly associated with decreased PFS time.

Conclusion: Baseline CMF and baseline AMC can be potential pan-cancer biomarkers to predict efficacy of PD-1/PD-L1 inhibitors, especially in the PD-L1 subgroup.

## Introduction

Programmed death 1 (PD-1, also called CD279) and its major ligand programmed death-ligand 1 (PD-L1, also called B7-H1 or CD274) consist a vital pathway to maintain peripheral tolerance [1]. PD-1 binds to PD-L1 on tumor cells or tumor infiltrating immune cells, and then facilitates cancer formation through inhibition of a cytotoxic T-cell response to avoid tumor cells from T cell cytolysis [2], which is a potent mechanism for tumors to escape from host immune responses. Preliminary clinical findings showed that blocker of PD-1 or PD-L1 was a promising therapy strategy [3], and was likely to enhance antitumor immunity with the potential to produce durable clinical responses [4]. Despite the encouraging results of previous clinical trials, the efficacy of PD-1/PD-L1 inhibitor alone is still restricted [5]. A series of clinical trials showed that the objective response rate (ORR) of PD-1/PD-L1 inhibitor in multiple tumor types was just between 15 and 26%, hardly exceeded 30% [6–14]. Besides, PD-1/PD-L1 inhibitor is expensive and has potential immune-related adverse events, which limited its routine clinical use. Therefore, reliable biomarkers to predict response before the initiation of treatment are urgently needed, for responsive patients to get immediate and effective treatment, and nonresponsive patients to adjust therapy strategy.

Recent studies have found that tumor mutational burden (TMB), PD-L1 expression of tumor tissue and immune cells, microsatellite instability (MSI), and mismatch repair (MMR) are all potential predictive biomarkers for PD-1/PD-L1 inhibitor. However, routine clinical use of these biomarkers is hampered by contradictory results of different studies, limited accessibility of patient material, lack of standardized methods, variable cut-off values, and the absence of independent cohorts for validation [15–18]. Therefore, there are still no perfect predictive biomarkers in clinical use up to date, and effort to explore new convenient, economical and effective biomarker is still a top issue. MSI-H/dMMR was the first pan-cancer predictive biomarker approved by Food and Drug Administration, though the positive rate among patients was rather low and not so effective in clinical use. The appearance of this issue showed that response of PD-1/PD-L1 inhibitor may no longer be restricted by tumor types, so pan-cancer predictive biomarkers were in need.

Monocyte in peripheral blood, especially classical monocyte, plays an important role in immune tolerance, and can differentiate into tumor-associated macrophage (TAM), which is abundant immune cell in the tumor stroma in a variety of cancers [19]. Studies showed TAM got divergent functions, but mostly appears as immunosuppression cells [20]. Therefore, monocyte especially its classical subtype may be potential predictive biomarkers of PD-1/PD-L1 inhibitor.

In the present study, we aimed to explore the efficacy of monocyte and its classical subtype in predicting response of PD-1/PD-L1 inhibitor. It is readily accessible, convenient to get assay results, and minimally invasive.

## Materials and methods

### Patient eligibility

Patients enrolled in our study were all from phase I trials of PD-1/PD-L1 inhibitors conducted in Fudan University Shanghai Cancer Center, and received a single PD-1/PD-L1 inhibitor treatment at dose of 1 mg/kg, 3 mg/kg, 10 mg/kg, 20 mg/kg, 200 mg, and 1200 mg. Eligible patients were aged ≥18 years and had histologically confirmed advanced or metastatic malignant cancer, with at least one measurable extracranial lesion based on RECIST Version 1·1. Any pre-treatment should be stopped at least 4 weeks (6 weeks for pre-immunotherapy) before first dose of study drugs. Patients in the present study had not responded to, and were unable to tolerate for standard treatment. Other inclusion criteria included an Eastern Cooperative Oncology Group (ECOG) 0–1 and adequate organ and marrow function, defined as absolute neutrophil count ≥ 1.5 × 10^9^/l, white blood cell ≥ 3.0 × 10^9^/l, platelet count ≥ 90 × 10^9^/l, hemoglobin ≥ 90 g/l, albumin ≥ 2.8 g/l, serum creatinine ≤ 1.5 upper limit of normal (ULN), ALT and AST ≤ 2.5 ULN (for patients with hepatic metastases, ≤ 5 ULN), total bilirubin ≤ 1.5 ULN (for patients with hepatic metastases, ≤ 2 ULN). Patients with central nervous system metastases were required to have had at least 4 weeks of nonprogression confirmed by CT or MRI. Exclusion criteria included patients with other malignant tumor in the past 5 years, except cured cervical carcinoma *in situ* or cured skin basal cell carcinoma; history of PD-1, PD-L1, or CTLA-4 treatment; history of immunodeficiency disease; history of autoimmune disease or uncontrolled systemic disease; active hepatitis A, B or C, and active tuberculosis; and history of transplant. prior severe or persistent immune-related adverse events. Patients who had not completed at least one response evaluation of PD-1/PD-L1 inhibitors were also excluded. The agents included HX008 (PD-1 inhibitor, Hanzhong Biomedical Co., Ltd, Taizhou, China), SHR1316 (PD-L1 inhibitor, Hengrui Medicine Co., Ltd, Jiangsu, China), CS1001 (PD-L1 inhibitor, CStone Pharmaceuticals Co., Ltd, Suzhou, China), ATEZOLIZUMAB (PD-L1 inhibitor, Roche Pharmaceuticals Co., Ltd, Shanghai, China), and LP002 (PD-L1 inhibitor, Houdeoke Technology Co., Ltd, Taizhou, China). Baseline clinical characteristics of each patient, such as sex, age, and metastasis sites were collected. Written informed consent was obtained from all patients before their enrollment in the trials.

The primary end point was response, which was evaluated by computed tomography of the chest, abdomen, and pelvis with or without brain magnetic resonance imaging every two cycles according to RECIST 1.1 criteria [21]. The benefit group comprised patients with complete response (CR), partial response (PR), and stable disease (SD), and the nonbenefit group included patients with progression disease (PD). The secondary end point was PFS, time interval from first dosage to observation of disease progression or any cause of death.

### Sample collection, AMC, and CMF testing

Baseline blood samples were collected from patients just before first dosage, and absolute monocyte count (AMC) was tested as part of the routine clinical procedure by a fully automated blood cell counting system (Sysmex 2100; Sysmex, Kobe, Japan) [22]. Peripheral blood mononuclear cells were isolated, and then percent of classical monocyte in the peripheral blood mononuclear cells was tested by flow cytometry, defined as classical monocyte frequency (CMF).

### Flow cytometry

In our study, classical monocyte was marked by CD14+ CD16− HLA-DR+ referred to previous study [15]. Peripheral blood mononuclear cells were isolated from freshly obtained EDTA-anti-coagulated whole blood samples according to Ficoll PBMC isolation protocols [23], and then stained with following murine anti-human monoclonal antibodies: 5 μl of anti-CD16 PE (Clone: B73.1, cat No.: 561313), 10 μl of anti-CD14 FITC (Clone: M5E2, cat No.: 555397), and 10 μl of anti-HLA-DR APC (Clone: TU36, cat No.: 559868) (all from BD Biosciences) and incubated for 30 min at room temperature in the dark. Next, cells were washed twice with cold phosphate-buffered saline and fixed with Cell Fix (BD Biosciences) [24]. Flow cytometry was performed using FC500 MPL software (Beckman Coulter) and data of CMF were analyzed using MXP Cytometer software (version 2.0, Beckman Coulter) (Supplementary Figure S1A,B).

### PD-L1 (TPS), TMB, MSI/MMR, EBER evaluation, and collection

In the present study, information of PD-L1 (TPS), TMB, MSI/MMR, and EBER was collected and analyzed. PD-L1 (TPS), MSI status, and TMB obtained from the medical test report card provided by Burning Rock Biotech (Guangzhou, China). The data of MMR and EBER were provided by the pathology department of our center. In brief, PD-L1 and MMR were assessed using immunohistochemistry according to previous study [25]. The monoclonal murine antihuman antibodies against PD-L1 (clone 22C3, Roche) were used. The expression of PD-L1 was evaluated by TPS score, which was described as 0–100% in the medical test report, and < 1% was defined as negative in the present study. TMB was tested by a commercially available 520-gene NGS panel in Burning Rock Biotech Limited as described previously [26], and cases were considered TMB-high if they had no less than 10 mutations each million bases, otherwise, it was defined as TMB-low. In the present study, cases with either MSI-H or dMMR were classified into MSI-H/dMMR group, and others were into MSI-L/MSS/pMMR group.

### Statistical analysis

Statistical analysis was carried out using GraphPad software (GraphPad Prism 6) and SPSS statistical software (version 22.0). Survival curves were created by the application of the Kaplan–Meier method, and the log-rank test was used to determine differences between survival proportions. Spearman correlation coefficient was used to determine correlations between variables. Statistical analysis between two groups was performed by *t* test, Chi-square test, and Fisher’s exact test. To assess the potential correlation between PFS and any of the clinical variables including AMC and CMF, we performed a Cox proportional-hazards regression. ROC curve was carried out using GraphPad software (GraphPad Prism 6). *P* values less than 0.05 were considered statistically significant.

## Results

### Patient characteristics

In total, 107 patients were enrolled in the present study, and the baseline characteristics of all evaluated patients were included in Supplementary Table S1. The most common tumor type was nasopharynx cancer (28 cases), breast cancer (12 cases), and esophageal cancer (10 cases) come next. Overall, the middle age at diagnosis was 51.9 (19–75) years, and more than half of patients were male (57.9%, *n*=62). PD-L1 expression of tumor tissue was evaluated in 32 patients, of whom 75% (24/32) patients had PD-L1 positive tumors. About 39 patients got MSI or MMR tested, and 20.5% (8/39) patients were MSI-H / dMMR. About 50 patients got EBER evaluated, and 38% (19/50) patients were positive. A total of 29 patients got TMB evaluation, and only 31% (9/29) patients were TMB-high. Detailed evaluation information of PD-L1 expression, MSI/MMR, EBER and TMB for each patient is shown in Supplementary Table S2. Among of 107 patients, 33 and 74 patients received PD-1 inhibitor and PD-L1 inhibitor alone treatment, respectively.

### Efficacy of PD-1/PD-L1 inhibitor

**Figure 1:**
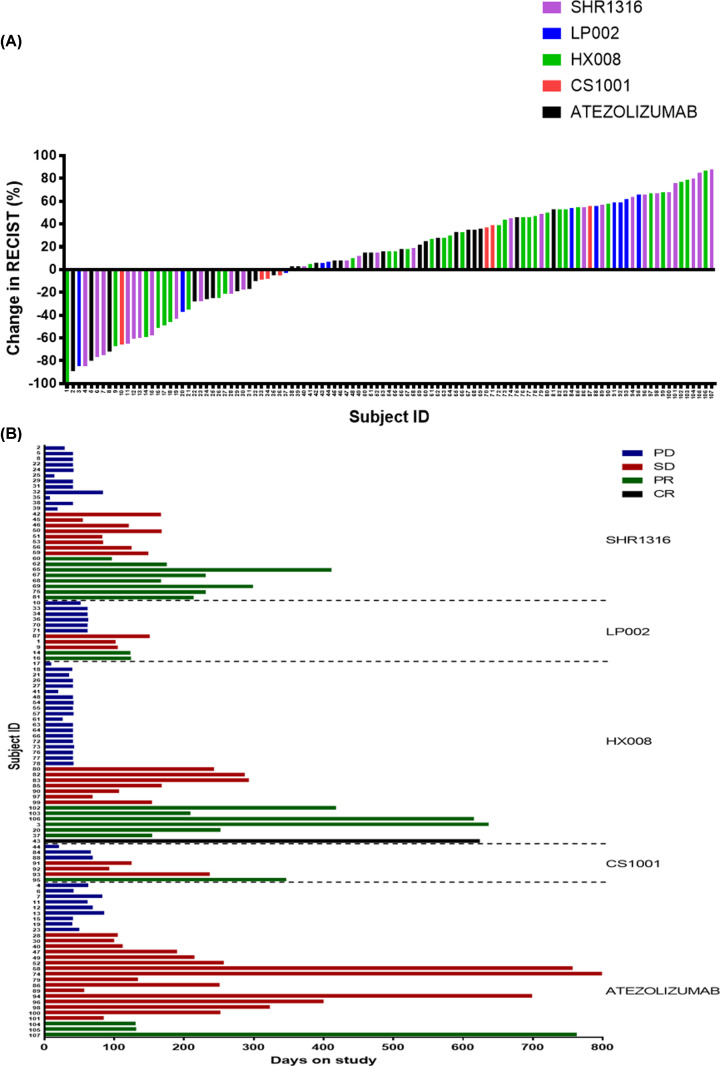
Response assessments (**A**) Waterfall plot. The columns are coded by agents: purple: SHR1316; blue: LP002; green: HX008; red: CS1001; black: ATEZOLIZUMAB. (**B**) Swimmers plot demonstrates times on study by PD-1/PD-L1 inhibitors. The bars are coded by best RECIST response: blue: PD; red: SD; green: PR; black: CR.

To explore efficacy, we examined response rate and PFS within different agent cohorts. At the end of follow-up, 1 (0.9%) patient diagnosed lymphoma had CR to HX008 (PD-1 inhibitor), 20 (18.7%) patients had PR to ATEZOLIZUMAB (PD-L1 inhibitor), CS1001 (PD-L1 inhibitor), HX008 (PD-1 inhibitor), LP002 (PD-L1 inhibitor), and SHR1316 (PD-L1 inhibitor), 37 (34.6%) patients had SD, and 49 (45.8%) patients had PD. Overall, the median PFS of all the 107 patients was 2.79 (0.26–26.25) months. In ATEZOLIZUMAB, CS1001, HX008, LP002, SHR1316 group, the median PFS was 3.99 (1.31–26.25), 3.05 (0.69–11.40), 1.38 (0.33–20.93), 2.07 (1.71–4.96), 2.76 (0.26–13.54) months, respectively. Figure 1A is a waterfall plot that depicts the best RECIST response in those who are eligible for response assessment. Figure 1B is a swimmers plot that groups patients by agents and depicts time on study and best response according to RECIST 1.1. In total, the ORR (CR, PR) of PD-1/PD-L1 inhibitor was 19.6% (21/107), and the clinical benefit rate (CBR) (CR, PR and SD) of PD-1/PD-L1 inhibitor was 54.2% (58/107). The number of CR, PR, SD, and PD in PD-1 inhibitor group was 1, 6, 7, 19, and in PD-L1 inhibitor group was 0, 14, 30, 30.

Response was examined within different agents and multicancer cohorts. The percentage of benefit group (CR, PR, and SD) to ATEZOLIZUMAB, CS1001, HX008, LP002, and SHR1316 was 67.9%, 57.1%, 42.4%, 45.5%, and 57.1% respectively (Supplementary Table S3). There was no significant difference among these groups (*P*=0.354).

Dose cohorts in our study consisted of 1 mg/kg, 3 mg/kg, 10 mg/kg, 20 mg/kg, 200 mg, and 1200 mg. The percentage of beneficiaries in these cohorts was 44.4%, 45.5%, 50.0%, 46.2%, 53.8%, and 65.7% respectively (Supplementary Table S4). There was no significant difference among response of patients treated with different dose levels (*P*=0.688).

### Baseline AMC may predict efficacy of PD-1/PD-L1 inhibitor

**Figure 2:**
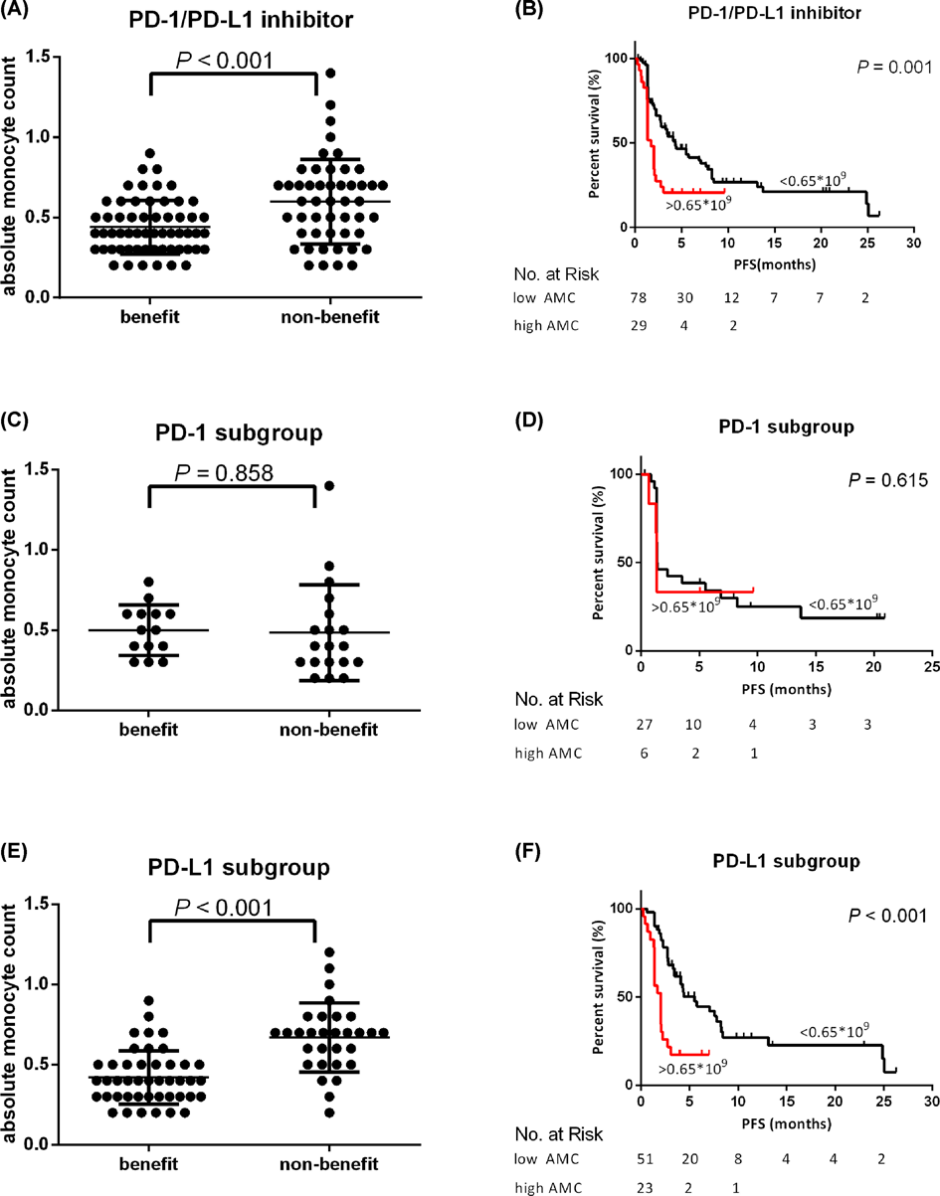
AMC predicts the efficacy of PD-1/PD-L1 inhibitors (**A**) The difference of AMC levels in benefit (*n*=58) and nonbenefit (*n*=49) group treated with PD-1/PD-L1 inhibitors. (**B**) Kaplan–Meier plot for PFS of patients with AMC high and low levels treated with PD-1/PD-L1 inhibitors. (**C**) The difference of AMC levels in benefit (*n*=14) and nonbenefit (*n*=19) group treated with PD-1 inhibitors. (**D**) Kaplan–Meier plot for PFS of patients with AMC high and low levels treated with PD-1 inhibitors. (**E**) The difference of AMC levels in benefit (*n*=44) and nonbenefit (*n*=30) group treated with PD-L1 inhibitors. (**F**) Kaplan–Meier plot for PFS of patients with AMC high and low levels treated with PD-L1 inhibitors.

In whole cohort, median AMC value was 0.50 × 10^9^/L ((0.50 ± 0.23) × 10^9^/L). Baseline AMC in benefit (CR, PR, and SD) and nonbenefit (PD) group was (0.44 ± 0.02) × 10^9^/L and (0.60 ± 0.04) × 10^9^/L, respectively. The benefit group had significantly lower AMC than the nonbenefit group (*P*<0.001) (Figure 2A).

ROC curve and Youden index model were performed, and 0.65 × 10^9^/L was established as the cut off to class the patients into AMC high and low groups (Supplementary Figure S2A). In which the sensitivity of AMC for predicting was 87.9% and the specificity was 44.9%.

The ORR of the patients in high AMC group was 10.3%, which had no significant difference from the low AMC group (23.1%), but showed a downward trend. CBR of high AMC group showed a notably lower than that of low AMC group (24.1% vs. 65.4%, *P*<0.001). The median PFS of patients in high AMC group was 1.71 months, which was significantly shorter than that of 4.30 months in low AMC group (*P*=0.001) (Figure 2B). In PD-1 group (*n*=33), the median AMC in beneficiaries was found no difference from non-beneficiaries ((0.50 ± 0.04) × 10^9^/L vs. (0.48 ± 0.07) × 10^9^/L, *P*=0.858) (Figure 2C), and also the median PFS in high AMC group had no significant difference compared with those of low AMC group (1.35 months vs. 1.40 months, *P*=0.615) (Figure 2D). In PD-L1 group (*n*=74), the beneficiaries had significantly lower AMC than nonbeneficiaries (0.42 ± 0.03 vs. 0.67 ± 0.04, *P*<0.001) (Figure 2E), and high AMC group had significantly shorter PFS than low AMC group (2.04 months vs. 5.52 months, *P*<0.001) (Figure 2F).

### CMF may predict efficacy of PD-1/PD-L1 inhibitor

**Figure 3:**
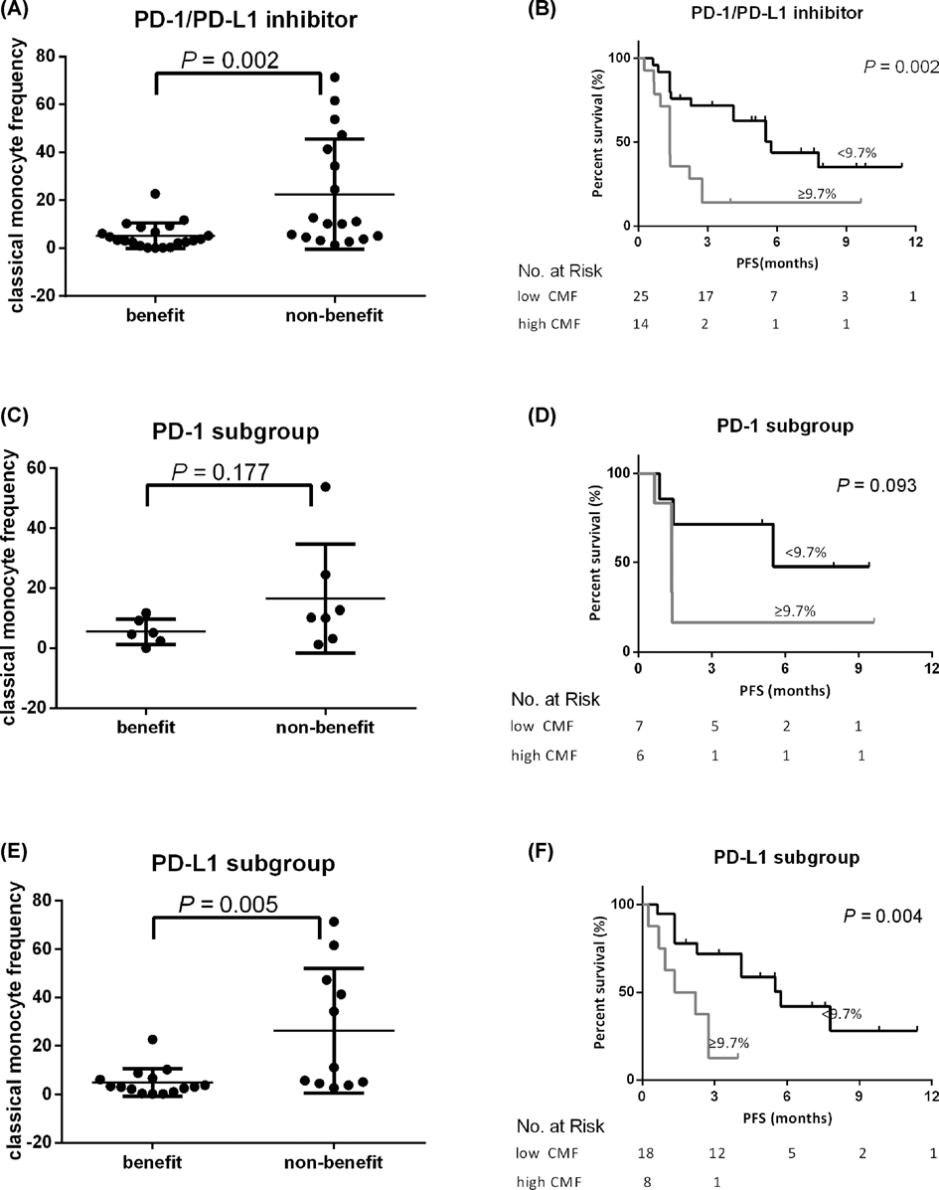
CMF predicts the efficacy of PD-1/PD-L1 inhibitors (**A**) The difference of CMF levels in benefit (*n*=21) and nonbenefit (*n*=18) group treated with PD-1/PD-L1 inhibitors. (**B**) Kaplan–Meier plot for PFS of patients with CMF high and low levels treated with PD-1/PD-L1 inhibitors. (**C**) The difference of CMF levels in benefit (*n*=6) and nonbenefit (*n*=7) group treated with PD-1 inhibitors. (**D**) Kaplan–Meier plot for PFS of patients with CMF high and low levels treated with PD-1 inhibitors. (**E**) The difference of CMF levels in benefit (*n*=15) and nonbenefit (*n*=11) group treated with PD-L1 inhibitors. (**F**) Kaplan–Meier plot for PFS of patients with CMF high and low levels treated with PD-L1 inhibitors.

Among all 107 patients, 39 had CMF tested in fresh blood samples and the median ratio of CMF was 5.1%. The benefit group had significantly lower CMF than the nonbenefit group (5.14% ± 1.16% vs. 22.48% ± 5.43%, *P*=0.002) (Figure 3A). According to ROC curve and Youden index, 9.7% was used to discriminate CMF from high and low groups with sensitivity of 85.7% for predicting response and specificity was 61.1% (Supplementary Figure S2B). Patients with CMF≥9.7% was CMF high group, and others were in CMF low group.

The ORR and CBR of patients with high CMF group were 0.0% and 21.4%, respectively, which was significantly lower than 32.0% and 72.0% of low CMF group (*P*=0.034 and 0.002, respectively). The median PFS of high CMF group was 1.35 months, which was significantly shorter than 5.75 months of low CMF group (*P*=0.002) (Figure 3B). In PD-1 group (*n*=13), the median CMF was 5.58% ± 1.75% in beneficiaries and 16.54% ± 6.83% in nonbeneficiaries, which had no significant difference (*P*=0.177) (Figure 3C). The PFS of high and low CMF groups were 1.3 and 5.2 months, respectively (Figure 3D). There was no significant difference in PFS between these two groups (*P*=0.093), but the PFS trend was longer in low CMF group. In PD-L1 group (*n*=26), the CMF of the beneficiaries was significantly lower than that of the non-beneficiaries (4.96% ± 1.49% vs. 26.26% ± 7.78%, *P*=0.005) (Figure 3E). The PFS of the low CMF group was 5.75 months, significantly longer than that of the high CMF group (1.78 months, *P*=0.004) (Figure 3F).

### In dependent predictive role of AMC and CMF to PD-1/PD-L1 inhibitors

To further explore the potential pan-cancer biomarkers to predict efficacy of PD-1/PD-L1 inhibitors, several classic biomarkers including tumor PD-L1 expression level, TMB, MSI/MMR, and EBER were assessed in the present study. We found that no relationship between response status and these biomarkers (Supplementary Table S5). The potential predictive roles of lymphocyte, leukocyte, and ratio of monocyte to lymphocyte/leukocyte were also assessed. The absolute lymphocyte and leukocyte count in benefit group was no significant different to the nonbenefit group (1.20 ± 0.07 vs. 1.21 ± 0.08, *P*=0.929; 5.79 ± 0.27 vs. 6.60 ± 0.35, *P*=0.065) (Supplementary Figure S3A,B). The ratio of monocytes to lymphocytes and to leukocytes in the benefit group were lower than that in the nonbenefit group (0.43 ± 0.03 vs. 0.57 ± 0.04, *P*=0.009; 0.08 ± 0.003 vs. 0.09 ± 0.004, *P*=0.016) (Supplementary Figure 3C,D). In univariate analysis model, the number of metastatic site (HR = 1.31, *P*=0.990), AMC (HR = 6.53, *P*=0.001), ratio of monocytes to leukocytes (HR = 5.7 × 10^4^, *P*=0.016) and CMF (HR = 1.03, *P*=0.001) but not the expression level of PD-L1 in the tumor (HR = 0.78, *P*=0.628), TMB (HR = 1.11, *P*=0.803), MSI/MMR (HR = 1.27, *P*=0.578), EBER (HR = 0.76, *P*=0.434), and ratio of monocytes to lymphocytes (HR = 1.40, *P*=0.302) were found to be significantly associated with PFS. In multivariate analysis model, only number of metastatic site (HR = 1.76, *P*=0.048), AMC (HR = 16.34, *P*=0.006), and CMF (HR = 1.04, *P*=0.002) were significantly associated with PFS (Table 1). Taken together, AMC and CMF had an independent predictive role to PD-1/PD-L1 inhibitors and could be used as a pan-cancer biomarker.

**Table 1:** Association of baseline clinical factors and progression free survival

Factors	Univariate analysis		Multivariate analysis	
	HR (95%CI)	*P* value	HR (95%CI)	*P* value
Sex	1.00 (0.64–1.56)	0.990		
Age	0.99 (0.97–1.01)	0.331		
Number of metastases	1.31 (1.01–1.70)	0.043	1.76 (1.01–3.07)	0.048
Visceral metstasis	1.38 (0.85–2.26)	0.193		
Dosage	1.00 (0.99–1.00)	0.349		
Tumor PD-L1 expression	0.78 (0.29–2.12)	0.628		
MSI/MMR	1.27 (0.55–2.96)	0.578		
EBER	0.76 (0.39–1.50)	0.434		
TMB	1.11 (0.48–2.61)	0.803		
AMC	6.53 (2.23–19.13)	0.001	16.34 (2.20–121.41)	0.006
lymphocyte	1.05 (0.70–1.58)	0.809		
M/Lym	1.40 (0.74–2.65)	0.302		
leukocyte	1.06 (0.96–1.17)	0.242		
M/Leu	5.7 × 10^4^ (7.54–4.4 × 10^8^)	0.016	0.0 (0.0–1.78 × 10^5^)	0.238
CMF	1.03 (1.02–1.05)	0.001	1.04 (1.01–1.06)	0.002

Abbreviations: AMC, absolute monocyte count; CMF, classical monocyte frequency; EBER, Epstein Barr encoded RNA; M/Leu, monocyte-to-leukocyte ratio; M/Lym, monocyte-to-lymphocyte ratio; MSI/MMR, microsatellite instability/mismatch repair; TMB, tumor mutational burden.

### Correlation between potential predictive factors

**Figure 4:**
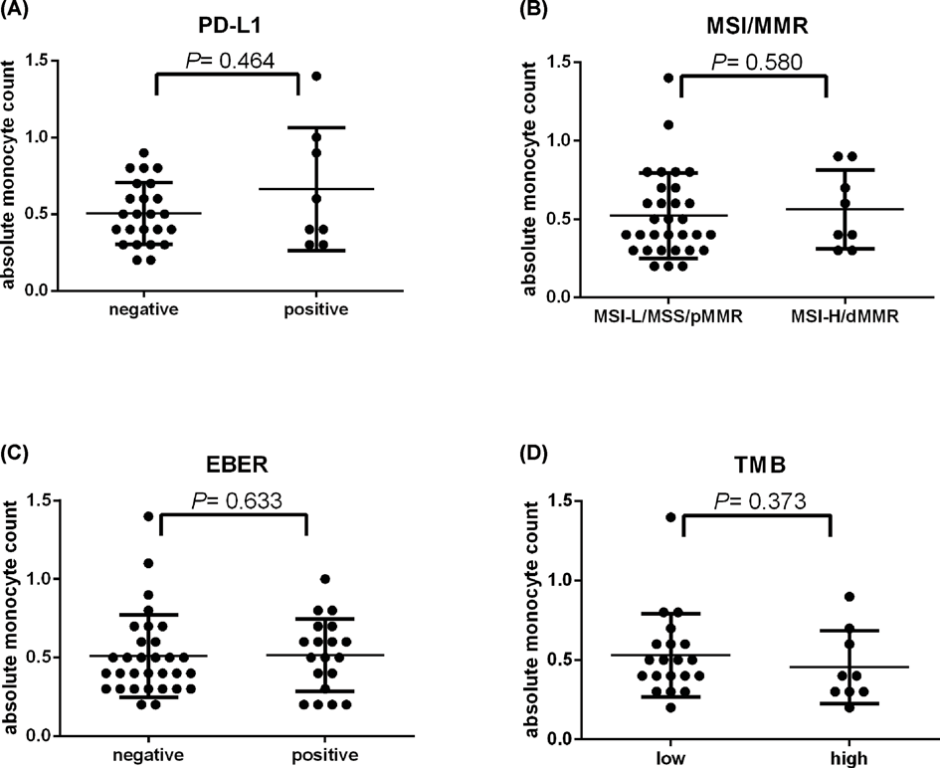
AMC has no relationship with PD-L1 (TPS), MSI/MMR, EBER and TMB AMC has no significant correlation with PD-L1 (TPS) (**A**) (*n*=32), MSI/MMR (**B**) (*n*=39), EBER (**C**) (*n*=50), and TMB (**D**) (*n*=29).

**Figure 5:**
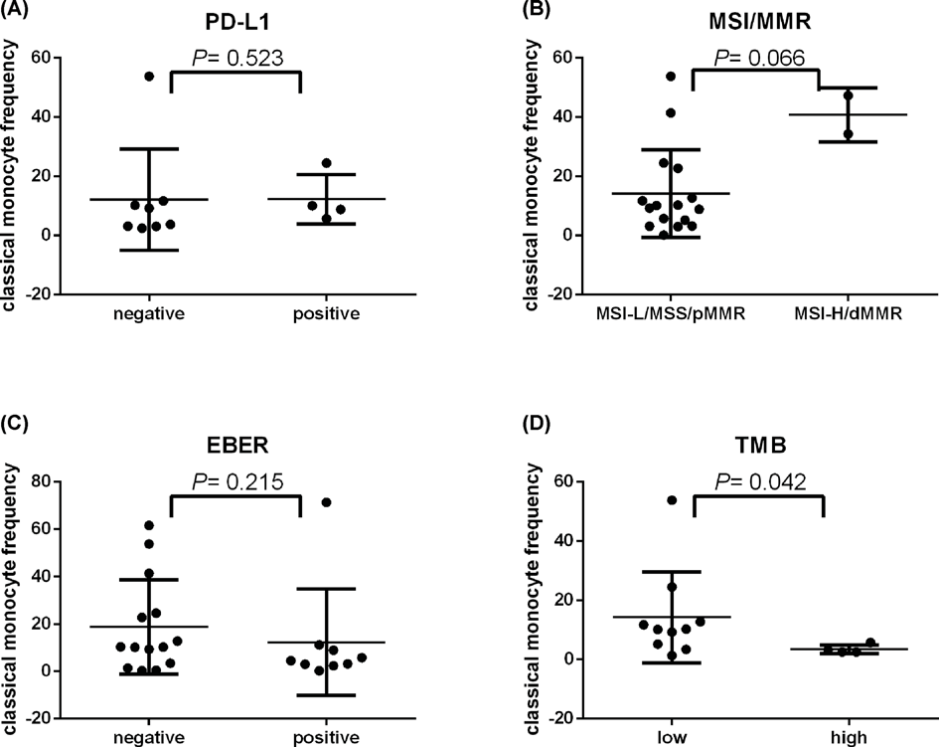
CMF is significantly associated with TMB CMF has no significant relationship with PD-L1 (TPS) (**A**) (*n*=12), MSI/MMR (**B**) (*n*=18), and EBER (**C**) (*n*=23), but has significant negative correlation with TMB (*P*=0.042) (**D**) (*n*=14).

We further evaluate the correlation among AMC, CMF, tumor PD-L1 expression, TMB, MSI/MMR, and EBER. We found that no significant relationship between tumor PD-L1 expression, MSI/MMR, EBER, and TMB with AMC (*P*=0.464, 0.580, 0.633, and 0.373, respectively) (Figure 4A–D). Similarly, tumor PD-L1 expression, MSI/MMR, and EBER also had no significant correlation with CMF (*P*=0.523, 0.066, and 0.215, respectively) (Figure 5A–C). However, TMB had significant negative correlation with CMF (Figure 5D), which means patients with high level of TMB often had lower CMF (*P*=0.042). Importantly, AMC had significant positive correlation with CMF (*r*=0.503, *P*=0.001).

### Examples of typical cases

**Figure 6:**
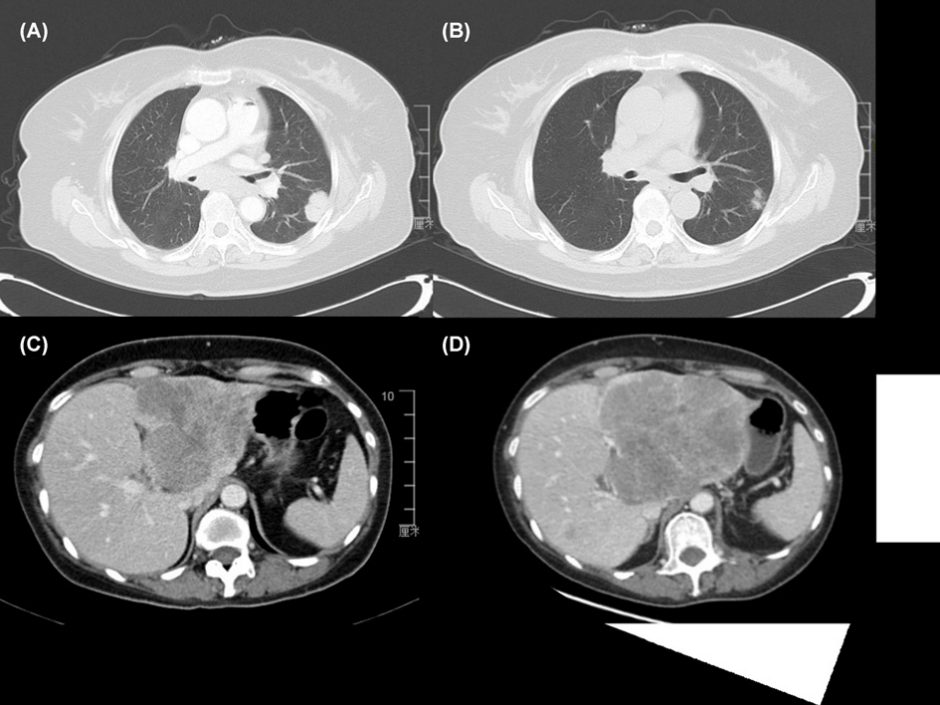
computed tomography images of typical cases The target lesion in lung at baseline was 29.1 mm (**A**) and decreased to 17.6 mm after six cycles in patient with nasopharyngeal cancer (**B**). The target lesion in liver at baseline was 98.5 mm (**C**) and progressed to 125.2 mm after two cycles in patient with rectal cancer (**D**).

Two representative cases were used to further demonstrate the predictive value of AMC and CMF for the efficacy of PD-1/PD-L1 inhibitors. The first one was nasopharyngeal cancer patient with lung metastases, and was treated with PD-L1 inhibitor (CS1001). The baseline AMC and CMF were 0.2 × 10^9^/L and 0.19%, respectively. After six cycles, the patient had a PR to the agent and has remained so far (Figure 6A,B). The other case was rectal cancer patient with liver and lung metastases, also was treated with PD-L1 inhibitor (CS1001). The baseline AMC and CMF were 0.9 × 10^9^/L and 34.3%, respectively. Just after two cycles, the patient developed PD to the agent (Figure 6C,D), despite diagnosis of MSI-H.

## Discussion

PD-1/PD-L1 inhibitor is one of the most promising therapies for cancers, but the efficacy is still limited. In our study, the ORR was 19.6%, and the CBR was 54.2%, consistent with most preliminary studies [6–10,12,27,28]. This result shows that most patients may not benefit from PD-1/PD-L1 inhibitor, and biomarkers for patient selection are urgent.

Monocytes are key components of the innate immune system and play a vital part in the maintenance of tissue homeostasis. Monocytes can stimulate or inhibit T-cell responses during cancer [29]. Chasseuil et al. [30] and Soyano et al. [31] found that increased AMC was significantly associated with decreased PFS time in melanoma and nonsmall cell lung cancer. Previous studies have also found that monocyte in peripheral blood and CMF may be potential predictive biomarkers for PD-1 inhibitor in melanoma [15,30], though the results are contradictory. In the present study, we found that low levels of baseline AMC and CMF were associated with better efficacy of PD-1/PD-L1 inhibitors in multiple cancers. Previous studies demonstrated that patients with high TMB tend to have better response of PD-1/PD-L1 inhibitors. We found that TMB was negatively associated with CMF. Taken together, monocyte and its classical subtype could be potential pan-cancer biomarkers of PD-1/PD-L1 inhibitors.

Monocytes, especially classical monocytes, in blood can infiltrate into local tissue and differentiate to macrophages, acting as immune-suppressive cells [20]. The potential mechanism of peripheral blood monocytes predicting the efficacy of immunotherapy may be related to the tissue infiltration and the differentiation to macrophages of monocytes. Shibutani et al. found that AMC was positively associated with the density of the TAMs [32]. Previous studies have revealed that high densities of TAM was significant associated with poor prognosis in numerous cancer types, including breast, thyroid, head and neck, liver, bladder, and so on [19]. Importantly, recent studies found that TAM could promote cancer immune evasion [33,34]. Some studies also found that in mice, many tumors selectively expanded classical monocytes, which seeded TAMs and promoted tumor growth [35]. Here, we come a hypothesis that monocytes translocation from peripheral blood to tissue induced a hot tumor in local tissue benefiting the PD-1/PD-L1 inhibitors treatment and decreased AMC and CMF in blood. This is consistent with our findings but need further evidence to support.

However, our study also has its limitations. First, patients included in our study were from different phase I studies, resulting in a diversity of cancer type, and in which several different investigational agents were given to patients at different dose levels. Although our and previously reported data [28,36] suggest that different doses have no significant effect on the efficacy of immunotherapy, the diversity of therapeutic agents is still an important confounding factor. Second, this was a retrospective analysis based on multiple Phase I studies, and too many confounding factors reduced the reliability of the study conclusions. The large number prospective trial is needed to further support our conclusion. Third, the number of patients who received test for TMB, MSI/MMR, EBER, and PD-L1 expression was relatively small and the test was nondefault. This could be the reason that we have not found the significant relationship between efficacy and these biomarkers which have been confirmed as biomarkers for predicting response of PD-1/PD-L1 inhibitor [17]. Last but not least, several factors including parasite or viral infection, autoimmune diseases, and dysfunction of bone marrow could affect the number of monocytes in the peripheral blood [37]. To eliminate the influence of the factors, we tried our best to exclude patients with these conditions in the present study.

In conclusion, our study is the first to show that monocyte and its classical subtype can be pan-cancer predictive biomarkers for PD-1/PD-L1 inhibitor, especially in PD-L1 subgroup.

## Perspectives

- PD-1/PD-L1 inhibitors have been promising treatment strategy in a variety of cancers, but there is a lack of pan-cancer predictive biomarker.
- We found that CMF and AMC are significantly associated with efficacy of PD-1/PD-L1 inhibitors. This suggests CMF and AMC can be potential pan-cancer biomarkers of PD-1/PD-L1 inhibitors.
- The noninvasive and repeatable properties of CMF and AMC make them more useful in predicting and dynamically monitoring the efficacy of immunotherapy.

## Supplementary Material

Supplementary Figures S1-S3 and Tables S1-S5

## Abbreviations

AMC: absolute monocyte count
CMF: classical monocyte frequency
EBER: Epstein Barr encoded RNA
M/Leu: monocyte-to-leukocyte ratio
M/Lym: monocyte-to-lymphocyte ratio
MSI/MMR: microsatellite instability/mismatch repair
PD: programmed death 1
PD-L1: programmed death-ligand 1
TAM: tumor-associated macrophage
TMB: tumor mutational burden
