# Supplementary material for: Baseline monocyte and its classical subtype may predict efficacy of PD-1/PD-L1 inhibitor in cancers
Source: Biosci Rep. 2021 Jan 29;41(1):BSR20202613. doi: 10.1042/BSR20202613 (PMC7846968; doi:10.1042/BSR20202613)
Supplement: Supplementary Figures S1-S3 and Tables S1-S5 [file BSR-2020-2613_supp.pdf]

Supplemental table 1 Baseline characteristics

Tumor type/site	Number	Sex		PD-L1		MSI/MMR		EBER		TMB		Agents				
		Male	Female	-	+	-	+	-	+	Low	High	A	C	L	S	H
nasopharynx	28	16	12	2	3	3	0	1	13	0	0	16	1	1	10	0
breast	12	0	12	3	1	6	0	7	1	4	1	0	0	4	2	6
esophagus	10	8	2	3	1	2	0	2	0	2	0	7	0	0	0	3
lymphoma	5	2	3	0	0	0	0	1	0	0	0	0	2	0	0	3
NSCLC	5	3	2	1	0	2	0	2	1	1	0	0	0	1	2	2
SCLC	1	0	1	0	0	0	0	0	0	0	0	0	0	0	1	0
melanoma	2	1	1	1	0	1	0	1	0	1	0	0	0	0	1	1
ICC	1	1	0	1	0	0	1	0	0	0	1	0	0	0	0	1
cervix uteri	3	0	3	1	0	0	0	1	0	0	0	0	0	0	3	0
colon	6	6	0	2	0	2	3	2	0	2	1	0	0	1	1	4
mesenchymal tumor	1	1	0	0	0	0	0	0	0	0	1	0	1	0	0	0
kidney	2	2	0	1	0	2	0	1	1	1	0	0	0	0	1	1
neuroendocrine	4	3	1	2	0	3	0	4	0	2	0	0	0	0	1	3
sarcoma	2	1	1	1	0	2	0	2	0	2	0	0	0	0	0	2
pancreas	3	2	1	0	1	1	1	2	0	2	0	0	1	0	0	2
CUP	2	2	0	0	1	1	0	0	2	0	1	0	0	0	2	0
rectum	5	3	2	2	0	3	1	0	0	1	2	0	1	1	0	3
HCC	4	4	0	0	1	0	0	0	0	0	0	4	0	0	0	0
larynx	2	2	0	1	0	0	0	0	0	0	0	0	0	1	1	0
stomach	2	1	1	1	0	1	0	1	1	1	0	1	0	0	0	1
ovary	2	0	2	1	0	0	1	2	0	0	1	0	0	0	1	1
hypopharynx	1	1	0	0	0	0	0	0	0	0	0	0	0	1	0	0
MPNST	1	1	0	0	0	1	0	1	0	1	0	0	0	0	1	0

**gallbladder**	1	1	0	0	0	1	0	0	0	0	0	0	1	0	0	0
**chordoma**	1	1	0	1	0	0	1	1	0	0	1	0	0	0	1	0
**endometrium**	1	0	1	0	0	0	0	0	0	0	0	0	0	1	0	0
**overall**	**107**	**62**	**45**	**24**	**8**	**31**	**8**	**31**	**19**	**20**	**9**	**28**	**7**	**11**	**28**	**33**

NSCLC: non-small cell lung cancer; SCLC: small cell lung cancer; ICC: intrahepatic cholangiocarcinoma; CUP: cancer of unknown primary; HCC: hepatocellular carcinoma; MPNST: malignant peripheral nerve sheath tumor; CR: complete response; PR: partial response; SD: stable disease; PD: progression disease; UK: response not evaluated yet; MSI/MMR: + means MSI-H or dMMR, **-** means MSS or MSI-L or pMMR; TMB: low means TMB **<** 10 mutations/Mb, high means TMB≥10 mutations /Mb; A: ATEZOLIZUMAB; C: CS1001; L: LP002; S: SHR1316; H: HX008.

Supplemental table 2 Detailed information of PD-L1, MSI/MMR, EBER and TMB

Number	Tumor type	PD-L1	MSI/MMR	EBER	TMB
1	nasopharynx	UK	UK	UK	UK
2	nasopharynx	UK	UK	negative	UK
3	nasopharynx	UK	UK	UK	UK
4	nasopharynx	UK	UK	positive	UK
5	nasopharynx	UK	low	positive	UK
6	nasopharynx	positive	low	positive	UK
7	nasopharynx	negative	low	positive	UK
8	nasopharynx	UK	UK	positive	UK
9	nasopharynx	UK	UK	UK	UK
10	nasopharynx	UK	UK	UK	UK
11	nasopharynx	UK	UK	UK	UK
12	nasopharynx	UK	UK	positive	UK
13	nasopharynx	negative	UK	positive	UK
14	nasopharynx	positive	UK	UK	UK
15	nasopharynx	UK	UK	UK	UK
16	nasopharynx	UK	UK	UK	UK
17	nasopharynx	UK	UK	positive	UK
18	nasopharynx	UK	UK	UK	UK
19	nasopharynx	UK	UK	UK	UK
20	nasopharynx	UK	UK	positive	UK
21	nasopharynx	UK	UK	UK	UK
22	nasopharynx	positive	UK	positive	UK
23	nasopharynx	UK	UK	UK	UK
24	nasopharynx	UK	UK	UK	UK
25	nasopharynx	UK	UK	positive	UK
26	nasopharynx	UK	UK	positive	UK
27	nasopharynx	UK	UK	positive	UK
28	nasopharynx	UK	UK	UK	UK
29	gallbladder	UK	low	UK	UK
30	melanoma	negative	low	negative	low
31	melanoma	UK	UK	UK	UK
32	MPNST	UK	low	negative	low
33	NSCLC	UK	UK	negative	UK
34	NSCLC	UK	UK	UK	UK
35	NSCLC	negative	low	positive	low
36	NSCLC	UK	UK	UK	UK
37	NSCLC	UK	low	negative	UK
38	SCLC	UK	UK	UK	UK
39	HCC	UK	UK	UK	UK
40	HCC	UK	UK	UK	UK
41	HCC	positive	UK	UK	UK
42	HCC	UK	UK	UK	UK
43	ICC	negative	high	UK	high
44	cervix uteri	negative	UK	UK	UK
45	cervix uteri	UK	UK	UK	UK
46	cervix uteri	UK	UK	negative	UK
47	larynx	UK	UK	UK	UK
48	larynx	negative	UK	UK	UK
49	chordoma	negative	high	negative	high
50	colon	UK	low	UK	low

51	colon	UK	high	UK	UK
52	colon	UK	UK	UK	UK
53	colon	UK	high	negative	UK
54	colon	negative	high	negative	high
55	colon	negative	low	UK	low
56	mesenchymal tumor	UK	UK	UK	high
57	lymphoma	UK	UK	negative	UK
58	lymphoma	UK	UK	UK	UK
59	lymphoma	UK	UK	UK	UK
60	lymphoma	UK	UK	UK	UK
61	lymphoma	UK	UK	UK	UK
62	ovary	UK	UK	negative	UK
63	ovary	negative	high	negative	high
64	sarcoma	UK	low	negative	low
65	sarcoma	negative	low	negative	low
66	breast	UK	low	negative	UK
67	breast	UK	UK	UK	UK
68	breast	negative	low	negative	high
69	breast	UK	UK	negative	low
70	breast	UK	UK	UK	UK
71	breast	negative	low	negative	low
72	breast	UK	low	positive	UK
73	breast	negative	low	negative	low
74	breast	positive	low	negative	low
75	breast	UK	UK	UK	UK
76	breast	UK	UK	UK	UK
77	breast	UK	UK	negative	UK
78	neuroendocrine	UK	low	negative	UK
79	neuroendocrine	negative	low	negative	low
80	neuroendocrine	UK	UK	negative	UK
81	neuroendocrine	negative	low	negative	low
82	kidney	negative	low	positive	low
83	kidney	UK	low	negative	UK
84	esophagus	UK	UK	UK	UK
85	esophagus	positive	UK	UK	UK
86	esophagus	UK	UK	UK	UK
87	esophagus	negative	UK	UK	UK
88	esophagus	UK	UK	UK	UK
89	esophagus	UK	UK	UK	UK
90	esophagus	UK	UK	UK	UK
91	esophagus	negative	low	negative	low
92	esophagus	UK	UK	UK	UK
93	esophagus	negative	low	negative	low
94	stomach	UK	UK	positive	UK
95	stomach	negative	low	negative	low
96	hypopharynx	UK	UK	UK	UK
97	pancreas	UK	high	UK	UK
98	pancreas	UK	UK	negative	low
99	pancreas	positive	low	negative	low
100	CUP	positive	low	positive	high
101	CUP	UK	UK	positive	UK
102	rectum	negative	low	UK	low
103	rectum	UK	high	UK	UK
104	rectum	UK	UK	UK	high
105	rectum	negative	low	UK	high

106	rectum	UK	low	UK	UK
107	endometrium	UK	UK	UK	UK

NSCLC: non-small cell lung cancer; SCLC: small cell lung cancer; ICC: intrahepatic cholangiocarcinoma; CUP: cancer of unknown primary; HCC: hepatocellular carcinoma; MPNST: malignant peripheral nerve sheath tumor; UK: results unknown

Supplemental table 3 Clinical benefit rate of agents

	ATEZOLIZUMAB	CS1001	HX008	LP002	SHR1316	*P*
**benefit**	19<br>67.9%	4<br>57.1%	14<br>42.4%	5<br>45.5%	16<br>57.1%	0.354
**non-benefit**	9<br>32.1%	3<br>42.9%	19<br>57.6%	6<br>54.5%	12<br>42.9%	

Supplemental table 4 Clinical benefit rate of different dose levels

	1mg/kg	3mg/kg	10mg/kg	20mg/kg	200mg	1200mg	*P*
**benefit**	4	5	13	6	7	23	0.688
	44.4%	45.5%	50.0%	46.2%	53.8%	65.7%	
**non-benefit**	5	6	13	7	6	12	
	55.6%	54.5%	50.0%	53.8%	46.2%	34.3%	

Supplemental table 5 Biomarkers assessment

parameters	Benefit	Non-benefit	*P* value
PD-L1 expression			1.00
positive	4	4	
negative	12	12	
MSI/MMR			0.697
MSI-H/dMMR	3	5	
MSI-L/MSS/pMMR	14	17	
EBER			0.608
positive	10	9	
negative	14	17	
TMB			0.822
≥10 mutations /Mb	4	5	
＜10 mutations/Mb	8	12	

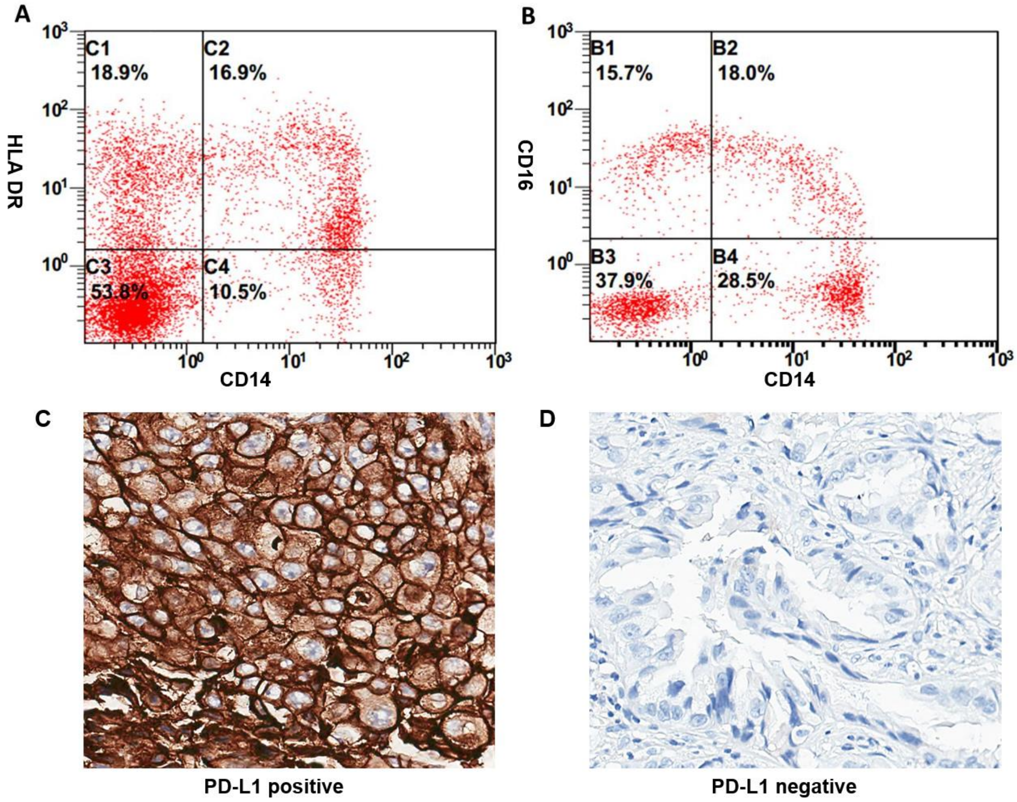

Supplemental figure 1 representative images for PD-L1 staining and flow cytometry

A: representative image of flow cytometry for gating strategy of CD14 and HLA DR. B: representative image of flow cytometry for gating strategy of CD14 and CD16. C: representative image of PD-L1 positive staining. D: representative image of PD-L1 negative staining.

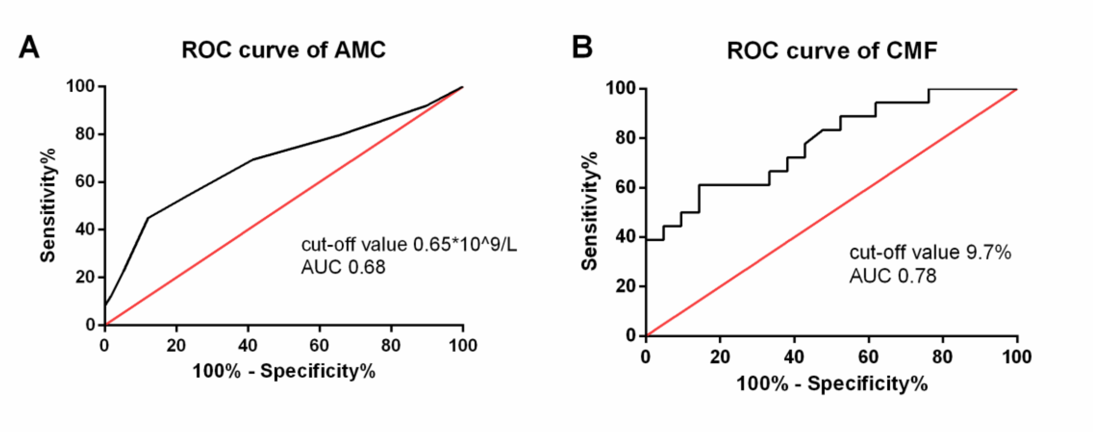

Supplemental figure 2 ROC curve of AMC and CMF

A: 0.65*10^9/L was the best cut-off value to class AMC into high and low groupwithsensitivity of 87.9% and specificity of 44.9%.B: 9.7% was used to discriminate CMF from high and low groups with area under curve of 0.78, the sensitivity of 85.7% and specificity of 61.1%.

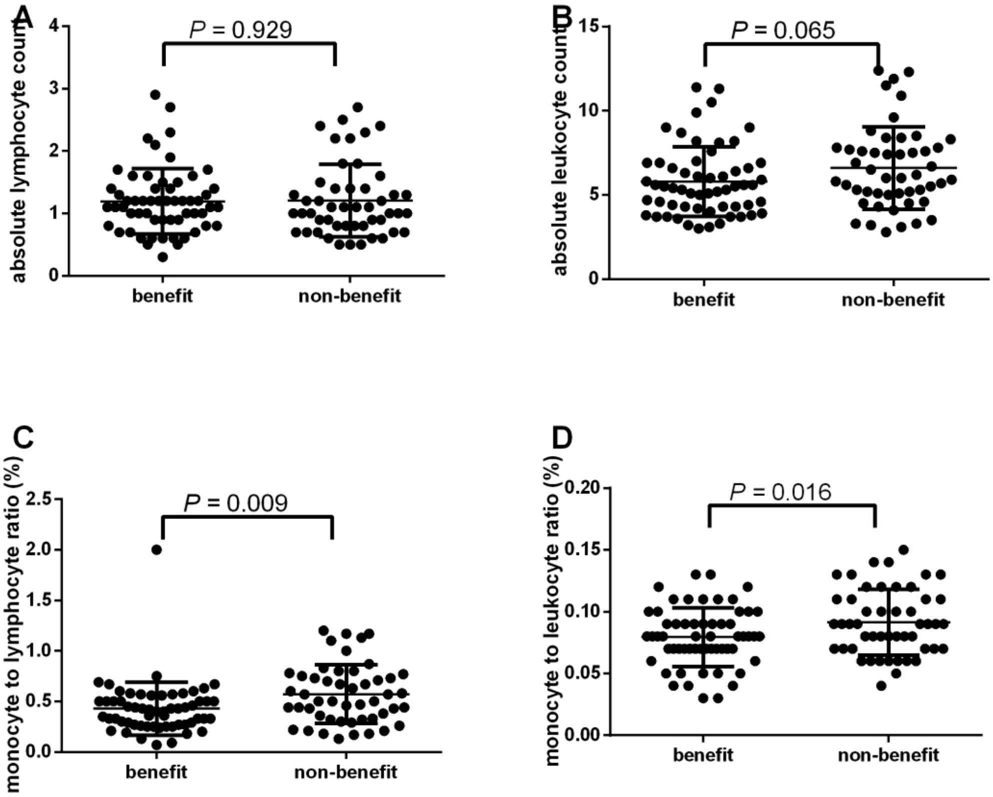

Supplemental figure 3 Lymphocyte and leukocyte assessment

A: There was no significant difference in the number of lymphocytes between benefit group (n=58) and non-benefit group (n=49) (*P*=0.929). B: There was no significant difference in the number of leukocytes between benefit group (n=58) and non-benefit group (n=49) (*P*=0.065). C: The benefit group (n=58) had significantly lower monocyte to lymphocyte ratio than the non-benefit group (n=49) (*P*=0.009). D: The benefit group (n=58) had significantly lower monocyte to leukocyte ratio than the non-benefit group (n=49) (*P*=0.016).
